# Correlating Pressure‐Induced Emission Modulation with Linker Rotation in a Photoluminescent MOF

**DOI:** 10.1002/anie.202000555

**Published:** 2020-03-17

**Authors:** Alif Sussardi, Claire L. Hobday, Ross J. Marshall, Ross S. Forgan, Anita C. Jones, Stephen A. Moggach

**Affiliations:** ^1^ EaStCHEM School of Chemistry The University of Edinburgh David Brewster Road Edinburgh EH9 3FJ UK; ^2^ WestCHEM School of Chemistry University of Glasgow University Avenue Glasgow G12 8QQ UK; ^3^ School of Molecular Sciences/ Centre for Microscopy, Characterisation and Analysis The University of Western Australia 35 Stirling Highway, Crawley Perth Western Australia 6009 Australia

**Keywords:** fluorescence, high-pressure chemistry, metal–organic frameworks, photophysics, UV/Vis spectroscopy

## Abstract

Conformational changes of linker units in metal‐organic frameworks (MOFs) are often responsible for gate‐opening phenomena in selective gas adsorption and stimuli‐responsive optical and electrical sensing behaviour. Herein, we show that pressure‐induced bathochromic shifts in both fluorescence emission and UV/Vis absorption spectra of a two‐fold interpenetrated Hf MOF, linked by 1,4‐phenylene‐bis(4‐ethynylbenzoate) ligands (**Hf‐peb**), are induced by rotation of the central phenyl ring of the linker, from a coplanar arrangement to a twisted, previously unseen conformer. Single‐crystal X‐ray diffraction, alongside in situ fluorescence and UV/Vis absorption spectroscopies, measured up to 2.1 GPa in a diamond anvil cell on single crystals, are in excellent agreement, correlating linker rotation with modulation of emission. Topologically isolating the 1,4‐phenylene‐bis(4‐ethynylbenzoate) units within a MOF facilitates concurrent structural and spectroscopic studies in the absence of intermolecular perturbation, allowing characterisation of the luminescence properties of a high‐energy, twisted conformation of the previously well‐studied chromophore. We expect the unique environment provided by network solids, and the capability of combining crystallographic and spectroscopic analysis, will greatly enhance understanding of luminescent molecules and lead to the development of novel sensors and adsorbents.

## Graphical abstract

**Pressure‐induced bathochromic shifts** in both fluorescence emission and UV/Vis absorption spectra of a two‐fold interpenetrated Hf MOF are induced by rotation of the central phenyl ring of the linker. Single‐crystal X‐ray diffraction was measured up to 2.1 GPa, thus correlating linker rotation with modulation of emission.

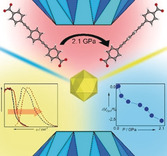

The high storage capacities and ease of functionalisation of metal‐organic frameworks (MOFs)^1^ has led to a significant body of work investigating their application as luminescent small‐molecule sensors.^2^ Anchoring organic chromophores as linkers within network solids can topologically isolate them from one another, negating any aggregation‐induced quenching processes^3^ and thereby maximising interactions between the host and the guest, although these sensing mechanisms are rarely structurally characterised. Dispersing discrete chromophores within a porous crystal also offers the possibility of spectroscopically studying what are effectively isolated molecules, with no significant intermolecular interactions to affect, for example, emission, whilst also collecting structural data crystallographically. Herein we demonstrate that both the structural and spectroscopic response to pressure of 1,4‐phenylene‐bis(4‐ethynylbenzoate) chromophores can be assessed, while bound within a Hf MOF, by diamond anvil cell (DAC) experiments.

DAC experiments in MOFs have become a favourable method for exploring mechanical stability,^4^ inducing ligand‐exchange reactions,^5^ locating gas molecules in the pores,^6^ causing changes in pore size and guest content,^7^ and even inducing low‐temperature melting of amorphous frameworks.^8^ These breakthroughs have been possible due to the way in which pressure is applied to these systems inside a DAC; crystals of the MOF are placed inside the cavity and surrounded with a liquid (hydrostatic) medium in order to apply pressure evenly to the sample. On increasing pressure to the MOF ZIF‐8, for example, this medium could be seen entering into the pores, causing the sample to expand, while at 1.47 GPa, the sample was observed to undergo a phase transition which resulted in a significant increase in pore volume and content.^9^ This transition was induced by a rotation of the imidazolate linkers on uptake of the hydrostatic medium into the pores at pressure, and is an example of ligand‐based flexibility, a characteristic of a plethora of MOFs that can be exploited to induce breathing mechanisms, gate opening gas adsorption, and phase transitions.^10^

More recently, high‐pressure studies on frameworks from the UiO‐family of MOFs, which consist of [M_6_O_4_(OH)_4_(RCO_2_)_12_] secondary building units (SBUs, M typically being Zr, Hf, Ce or Ln) linked by organic dicarboxylates, have been found to have excellent stability towards extremes of pressure.^11^ The behaviour of Zr MOFs linked by 4,4′‐biphenyldicarboxylate (bpdc) and 4,4′‐azobenzenedicarboxylate (abdc) linkers, referred to as UiO‐67(Zr) and UiO‐abdc(Zr), respectively, was heavily dependent on the linker and the hydrostatic liquid used during the pressure experiment. For example, when crystals of both UiO‐67(Zr) and UiO‐abdc(Zr) were compressed using methanol as a hydrostatic liquid, the crystals were essentially incompressible; however, when compressed using large perfluorinated oils (that were too large to penetrate the pores), the crystals showed direct compression. Surprisingly, the MOF composed of the longer organic linkers (abdc) was more resilient to direct compression of the framework, and pressure compliance was attributed to framework‐dynamic behaviour, much like a suspension bridge, whereby some flexibility in the linking struts gives rise to a more stable structure.^12^ Similarly, recent work by Suslick et al., has shown that UiO‐66(Zr), which has shorter terephthalate linkers, undergoes bond‐breakage under pressure, and is even less stable.^13^ Ligand‐flexibility in UiO‐MOFs can also be induced by guest inclusion, leading to changes in fluorescence emission spectra, with potential for use of these materials as sensors.^14^ The intrinsic fluorescence of Zr‐MOFs has also been demonstrated to be useful in pH sensing,^15^ while changes in ligand conformation in tetraphenylethylene‐16 and porphyrin‐linked^17^ Zr MOFs have been shown to result in differences in their steady‐state emission spectra, although there is no in situ X‐ray diffraction data to provide structural mechanisms for such changes on uptake of guest molecules.

In this study we present a combined high‐pressure diffraction and spectroscopic study on a Hf‐UiO‐MOF connected by the longer 1,4,‐phenylene‐bis(4‐ethynylbenzoate) linker (peb^2−^), subsequently designated **Hf‐peb** for simplicity. High‐pressure diffraction data were collected up to 2.1 GPa, using a modified Merrill‐Bassett DAC (see ESI section 1), whilst corresponding in situ measurements of UV‐vis absorption and fluorescence emission spectra were made in the same DAC, to compare directly to the diffraction data (ESI sections 2 and 3).

We have previously reported the synthesis and crystal structure of **Hf‐peb**,^18^ which crystallises in the cubic crystal system in space group $Fd\bar{3}m$ , and consists of Hf_6_O_4_(OH)_4_ SBUs, as seen in the zirconium MOFs UiO‐67(Zr) and UiO‐abdc(Zr). The longer linker gives rise to two‐fold interpenetration (Figure 1 a); MOFs of the isoreticular Zr series were originally described as PIZOFs (porous interpenetrated zirconium organic frameworks).^19^ Under ambient pressure and temperature conditions the peb^2−^ has a non‐planar “bowed” geometry in the crystal structure (Figure 1 b), with significant libration occurring within the ligand (Figure S1 & S2). This is unsurprising for these compounds, where ab initio calculations have previously shed light on similar movement of such ligands.^11^, ^20^

**Figure 1:**
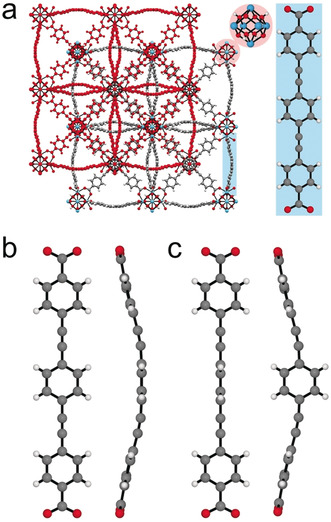
a) The solid‐state structure of **Hf‐peb**, showing the Hf_6_ SBU and the planar conformation of the peb^2−^ ligand. Structures of b) the coplanar and c) the twisted conformers of the peb^2−^ linker, viewed perpendicular and parallel to the linker plane.

Under ambient temperature and pressure conditions, the central phenyl ring of the peb^2−^ ligand was found to be disordered over two positions, one being coplanar with the other two phenyl rings (and the rest of the peb^2−^ ligand), and the other perpendicular to them. For simplicity, we refer to these two geometries as coplanar and twisted (Figure 1 b and c). The two conformers are not equally populated, with the coplanar structure constituting 73 % of the population under ambient pressure at room temperature. This corresponds to a difference in energy of 2.5 kJ mol^−1^ between the two conformers, which is close to the value of 2.7 kJ mol^−1^ reported for the barrier to torsion of the central ring in the free 1,4‐bis(phenylethynyl)benzene (BPEB) molecule,^21^ where the twisted structure is the transition state on the torsional potential. In our previously reported structure of **Hf‐peb**, (and the structures of the Zr analogues)^14^, 19a such disorder was not observed, but these data were collected at low temperature (150 K), where the twisted conformer was not populated.^14^

On increasing pressure, the population of the twisted conformer increased, reaching 100 % occupancy at 2.1 GPa (Figure 2 a). A small expansion in volume was observed on loading the crystal into the DAC at 0.1 GPa (39 Å^3^, 0.006 %), although the change observed was small and barely significant (3.2σ). The unit cell volume then decreased with increasing pressure (Figure 2 b), with a reduction of ≈2.6 % at 2.1 GPa, a resulting reduction in the crystallographic *a*‐axis by 0.349(2) Å. Interestingly, on increasing pressure above 1.1 GPa, a discontinuity in the unit cell volume was observed. This discontinuity may be caused by a subtle degree of bending observed for the peb^2−^ ligand, which distorts to allow the Hf_6_ SBUs to compress closer together, however the differences observed here are marginal, and more data points as a function of pressure would be needed to confirm this. Nevertheless, the overall bending observed across the entire pressure study here, is analogous to the motion which stabilises UiO‐abdc(Zr) to increased pressures, and can be quantified by measuring the distance between the carboxylate C‐atoms in the peb^2−^ ligand as a function of pressure (*d*_CO_ in Figure 2 c), where the decrease in distance matches the compressibility of volume (Figure 2 b). This plateau also coincides with a sudden increase in population of the twisted conformer above 1.1 GPa (Table S1 and Figure 2 a). In summary, we see an increase in the occupancy of the twisted conformer on increasing pressure, with a sharp increase in population above 1.1 GPa, which corresponds with the decreasing trend in *d*_CO_. It would appear, therefore, that a decrease in population of the planar conformer gives rise to a less rigid and more flexible framework. In addition, both of these effects appear to actually increase the pore volume, with an increase of 1105 Å^3^ on increasing the pressure from ambient to 2.1 GPa (Table S2). This is similar to behaviour observed on increasing pressure in other MOFs, such as ZIF‐8,^22^ HKUST‐1,7b, 23 Sc_2_BDC_3_ 7a and ZAG‐based MOFs,^24^ where ligand flexibility facilitates significant pore volume and content changes, though the changes observed here are much smaller, and the mechanism (i.e. increase in population of a conformer that is less favourable under ambient conditions), is completely new.

**Figure 2:**
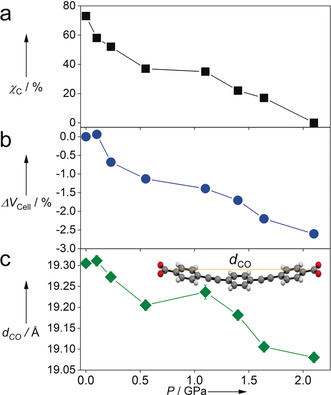
The responses of a) the fractional population of the coplanar structure, *χ*_C_, obtained from occupancy refinement of the X‐ray data, b) unit cell volume, and c) peb^2−^ length, *d*_CO_, to increasing pressure. Error bars are not shown in (a) and (b), as they are only marginally larger, or even smaller, than the size of the points.

In our previous work, large changes in the fluorescence emission spectra on increasing relative humidity were observed for analogous Zr UiO‐MOFs with naphthyl‐ and benzothiadiazolyl‐modified peb^2−^ linkers.^14^ Here, in order to directly determine structure/property relationships in this class of porous framework, we collected absorption and emission spectra of a crystal of **Hf‐peb** as a function of increasing pressure to 2.1 GPa, using custom built UV/Vis absorption and fluorescence emission spectrometers (Figure 3 a, ESI sections 2 and 3). Both the UV/Vis absorption spectrum and the fluorescence emission spectrum shift to longer wavelengths on increasing pressure (Figure 3 b and c, Figures S5 and S9, Tables S5 and S7). The pressure‐induced bathochromic shift of the emission spectrum is much greater than that of the absorption spectrum, reflecting structural relaxation in the excited state, which is discussed below.

**Figure 3:**
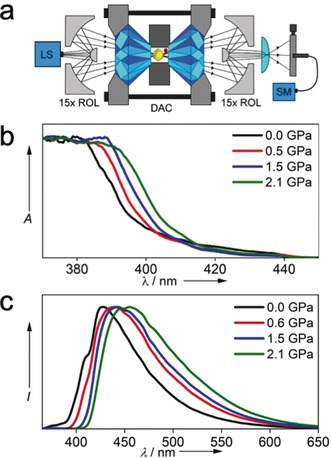
a) Experimental system for in situ measurement of fluorescence spectra within a diamond anvil cell. (LED LS: light source; ROL: reflective objective lens; DAC: diamond anvil cell; SM: fibre‐coupled spectrometer) b) Pressure‐dependence of the UV/Vis absorption spectrum. (Absorbance, A, is normalised). c) Pressure‐dependence of the fluorescence spectrum, at an excitation wavelength of 380 nm. (Fluorescence intensity, I, is normalised).

It was found that the observed emission spectra of **Hf‐peb**, across the pressure range, could be well‐fitted by linear combinations of the 2.1 GPa spectrum, assigned to the twisted conformer, and the ambient pressure spectrum (ESI Section 2.2). On this basis, the fractional population of the twisted conformer at each pressure was estimated (Table S6) and found to be in good agreement with the values determined from the X‐ray diffraction data (Figure 4). This close correlation comes from measurements on two separate crystals, indicating these effects are not crystal dependent. The close correlation indicates that the contribution of each conformer to the observed emission spectrum is determined by its ground‐state population, and hence the two conformers must have very similar fluorescence brightness (the product of the molar absorption coefficient, at the excitation wavelength (380 nm), and the fluorescence quantum yield). The brightness and the emission spectral profile of each conformer must also be essentially independent of pressure. Based on the spectral fitting we can therefore extract the fluorescence spectrum of the coplanar conformer (Figure S7), which has its maximum at 424 nm, compared with 450 nm for the twisted form. The twisted conformer shows a significantly greater Stokes shift than the coplanar form (2830 cm^−1^ compared with 1750 cm^−1^) and its emission profile is broader.

**Figure 4:**
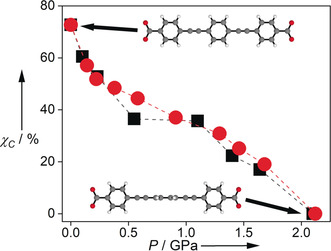
The fractional population of the coplanar structure, *χ*_C_, obtained from occupancy refinement of the X‐ray data (black squares) and from fitting the fluorescence spectra (red circles). Lines are for guidance only.

This indicates that, following excitation of the twisted conformer to the Franck‐Condon geometry, there is structural relaxation prior to emission, and the extent of this relaxation is greater for the twisted conformer than for the coplanar one. This is consistent with previous molecular orbital calculations on the free BPEB molecule, which show that the excited‐state electronic structure of the twisted form differs substantially from that of the ground state, adopting a more localised quinoid structure, while the excited state of the coplanar form retains the delocalised benzenoid structure of the ground state.^25^ In **Hf‐peb**, upon excitation of the twisted conformer, the local MOF structure responds to the change in electronic structure of the ligand. This is a further illustration of the flexibility of the MOF framework, and how this can influence the photoluminescence properties.^26^ This behaviour is in contrast to a recently reported two‐fold interpenetrated tetraphenylethylene‐based Zr MOF, which showed changes in luminescence when pressurised up to 20 MPa as a consequence of a breathing effect, whereby the two interpenetrating nets were compressed towards one another and the unit cell contracted dramatically.^27^ Finally, on decreasing pressure, the pressure‐induced spectral shifts were reversible, indicating the transition from a mixed twisted:planar structure at ambient pressure, to a 100 % twisted structure at 2.1 GPa, is fully reversible.

In summary, we have collected high‐pressure diffraction data, UV/Vis absorption and fluorescence emission spectra of the UiO‐MOF **Hf‐peb** to 2.1 GPa. The MOF undergoes a reversible phase transition on increasing pressure, where the ligand conformation changes from coplanar to twisted. This transition gives rise to both a shift in the UV/Vis absorption and fluorescence emission spectra, with the latter being influenced by relaxation of the MOF structure around the excited ligand. Our ability to directly correlate X‐ray structures and optical spectra has shown that the observed bathochromic shift in emission is not caused by the pressure‐induced shift in excitation energy of a single emitting species, but by the interconversion of two spectroscopically distinct ground‐state species whose emission spectra are essentially independent of pressure. Given that previous work on Zr‐based MOFs containing the peb^2−^ ligand has shown that the optical properties of the MOF are inherited from the π‐conjugated organic ligand,^28^ it would appear that observation of the spectroscopic response to structural changes within the MOF is an ideal approach for studying, in a controlled fashion, the photophysics of the linkers, by enabling their entrapment in the framework and subsequent pressure‐ and guest‐induced conformational changes to be followed. We also aim to determine the reasons for the transition occuring in the first place (i.e. is it caused by host‐guest interactions, or simply by pressure itself). We then aim to subsequently apply these methods to similar systems where the ligand can be functionalised appropriately to test these theories. We therefore believe that we are now in a position to determine systematically the optimum properties for maximizing fluorescence emission changes, and thereby advance the development of these materials for sensing and photocatalytic applications.

## Conflict of interest

The authors declare no conflict of interest.

## Supporting information

As a service to our authors and readers, this journal provides supporting information supplied by the authors. Such materials are peer reviewed and may be re‐organized for online delivery, but are not copy‐edited or typeset. Technical support issues arising from supporting information (other than missing files) should be addressed to the authors.

Supplementary
