# Supplementary material for: Correlating Pressure‐Induced Emission Modulation with Linker Rotation in a Photoluminescent MOF
Source: Angew Chem Int Ed Engl. 2020 Mar 17;59(21):8118–22. doi: 10.1002/anie.202000555 (PMC7317771; doi:10.1002/anie.202000555)
Supplement: Supplementary file 1 — Supplementary [file ANIE-59-8118-s001.pdf]

Angewandte Chemie
Eine Zeitschrift der Gesellschaft Deutscher Chemiker

Supporting Information

Correlating Pressure-Induced Emission Modulation with Linker Rotation in a Photoluminescent MOF

Alif Sussardi, Claire L. Hobday, Ross J. Marshall, Ross S. Forgan, Anita C. Jones,* and Stephen A. Moggach**

anie_202000555_sm_miscellaneous_information.pdf

1. Crystallography

1.1 High-pressure single-crystal X-ray diffraction

A single crystal of Hf-peb (peb = 4,4'-[1, 4,-phenylene-bis(ethyne-2,1-diyl)]benzoate)) was loaded into a Merrill-Bassett diamond anvil cell with a half-opening angle of 40 degrees, composed of Boehlar Almax diamonds with 600µm culet diamond anvils, a tungsten gasket and tungsten carbide backing plates.[1] A small ruby chip was also loaded into the cell to act as an internal pressure calibrant, using the pressure-dependent fluorescence of the ruby to measure the pressure,[2] while pentane was added as a pressure transmitting medium (PTM). Diffraction data were collected on a Bruker Apex II diffractometer and CCD detector using Mo-Kα radiation (λ = 0.71073 Å). Data collections were carried out using an exposure time and a step size of 1 second and 0.5 degrees respectively. The data were integrated using the programme SAINT using dynamic masks, these mask the regions of the detector which are shaded due to the pressure cell. The omission of shaded reflections, absorption correction and merging of data were carried out in a three-step process, firstly with the programme SHADE, then SADABS and finally XPREP.[3] Crystallographic data for the structures reported in this paper can be found in the Supporting Information of this paper and have been deposited at the Cambridge Crystallographic Data Centre (CCDC 1976393-1976400). These can be obtained free of charge via https://www.ccdc.cam.ac.uk/structures/, or from the Cambridge Crystallographic Data Centre, 12 Union Road, Cambridge CB2 1EZ, U.KCB21EZ, UK (fax +441223336033; email deposit@ccdc.cam.ac.uk).

1.2 Structure refinements

Structure refinements were carried out in CRYSTALS.[4] All structures were refined against F^2. All 1,2 and 1,3 distances for the organic linker were restrained, whilst all torsion angles and metal-ligand bond distances were allowed to freely refine. Vibrational and thermal similarity restraints were applied to the organic linker and based on the measurements collected under ambient conditions due to the significant libration of the ligand (Figure S1). Hydrogen atoms on the linker were placed geometrically and constrained to ride on their host atoms. The pore volume and electron count per unit cell (and therefore the solvent count) were calculated using the SQUEEZE algorithm in PLATON (Table S1).

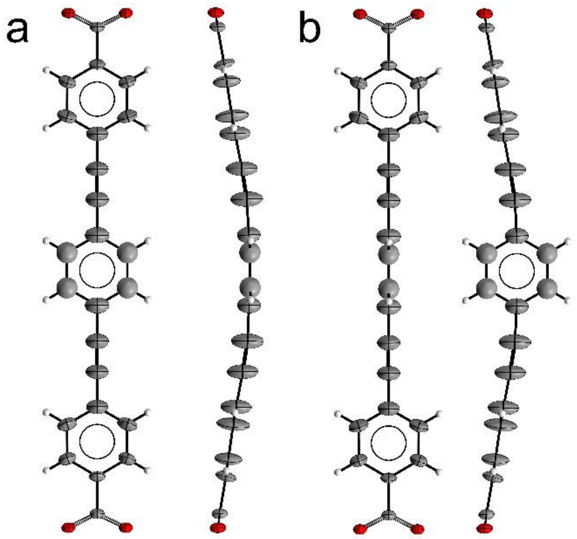

Figure S1. The coplanar (a) and twisted (b) conformers of the peb^{2-} linker, viewed perpendicular and parallel to the linker plane. Anisotropic displacement parameters are drawn at 30% probability for all but the central phenyl ring.

Table S1. Fractional population of coplanar (χ_C) and twisted (χ_T) conformers as a function of pressure, from x-ray data.

Pressure (GPa)	χ_C	χ_T
**0.00**	0.727(10)	0.273(10)
**0.10**	0.580(15)	0.420(15)
**0.23**	0.520(14)	0.480(14)
**0.55**	0.365(18)	0.635(18)
**1.10**	0.350(20)	0.650(20)
**1.40**	0.221(16)	0.779(16)
**1.64**	0.172(12)	0.828(12)
**2.10**	0.00	1.000

Table S2. Platon output using the SQUEEZE algorithm. Pore content and volume were calculated using a step size and probe-radius of 0.2 and 1.*2* Å respectively.

Pressure (GPa)	Pore volume (*Å³*)	Pore content (electrons)
**ambient**	42111	21293
**0.10**	41967	9180
**0.23**	41823	6570
**0.55**	41906	7199
**1.10**	41886	8088
**1.40**	42356	7347
**1.64**	43471	8296
**2.10**	43216	8449

Table S3. d_{CO} distances as a function of pressure. Note that the distance quoted is from the carboxylate C-atom.

Pressure (GPa)	d_{CO} length(*Å*)
**ambient**	19.305(9)
**0.10**	19.311(13)
**0.23**	19.272(12)
**0.55**	19.205(13)
**1.10**	19.236(17)
**1.40**	19.181(13)
**1.64**	19.106(13)
**2.10**	19.080(13)

Table S4: Abbreviated crystallographic tables for all structures: M_r = 1694.55, Cubic, $Fd\bar{3}m$, Z = 16. Experiments were carried out with Mo *K*a radiation using a Bruker Kappa Apex2. Absorption was corrected for by multi-scan methods, *SADABS* (Siemens, 1996).

Pressure / GPa	Ambient	0.10	0.23	0.55
Crystal data				
Chemical formula	$C_{72}H_{38}Hf_3O_{16}$	$C_{72}H_{38}Hf_3O_{16}$	$C_{72}H_{38}Hf_3O_{16}$	$C_{72}H_{38}Hf_3O_{16}$
Temperature (K)	299	300	296	296
a (Å)	39.8212 (18)	39.8295 (17)	39.7307 (8)	39.6707 (9)
V (Å^3)	63146 (9)	63185 (8)	62716 (4)	62432 (4)
m (mm^{-1})	2.00	2.00	2.01	2.02
Crystal size (mm)	0.10 × 0.10 × 0.10	0.10 × 0.10 × 0.10	0.10 × 0.10 × 0.10	0.10 × 0.10 × 0.10
Data collection				
T_{min}, T_{max}	0.67, 0.82	0.36, 0.82	0.42, 0.82	0.47, 0.82
No. of measured, independent and observed [I > 2.0s(I)] reflections	94905, 1635, 1287	48173, 1700, 1111	43461, 1481, 988	42979, 1490, 991
R_{int}	0.057	0.094	0.087	0.083
q_{max} (°)	20.9	22.0	20.8	20.9
(sin q/l)$_{max}$ (Å^{-1})	0.503	0.527	0.500	0.501
Refinement				
$R[F^2 > 2s(F^2)]$, $wR(F^2)$, S	0.033, 0.069, 1.05	0.038, 0.080, 1.00	0.042, 0.114, 1.01	0.043, 0.130, 1.00
No. of reflections	1593	1627	1410	1431
No. of parameters	91	90	1	91
No. of restraints	139	133	0	134
H-atom treatment	H atoms treated by a mixture of independent and constrained refinement	H atoms treated by a mixture of independent and constrained refinement	H-atom parameters not refined	H atoms treated by a mixture of independent and constrained refinement
Dρ_{max}, Dρ_{min} (e Å^{-3})	0.64, -1.02	1.13, -0.91	1.63, -0.80	1.84, -0.88

Pressure / GPa	1.10	1.40	1.64	2.10
Crystal data				
Chemical formula	$C_{72}H_{38}Hf_3O_{16}$	$C_{72}H_{38}Hf_3O_{16}$	$C_{72}H_{38}Hf_3O_{16}$	$C_{72}H_{38}Hf_3O_{16}$
Temperature (K)	296	296	296	296
a (Å)	39.6359 (10)	39.5937 (7)	39.5272 (7)	39.4727 (8)
V (Å^3)	62268 (5)	62070 (3)	61757 (3)	61502 (4)
m (mm^{-1})	2.02	2.03	2.04	2.05
Crystal size (mm)	0.10 × 0.10 × 0.10	0.10 × 0.10 × 0.10	0.10 × 0.10 × 0.10	0.10 × 0.10 × 0.10
Data collection				
T_{min}, T_{max}	0.43, 0.82	0.47, 0.82	0.49, 0.82	0.49, 0.81
No. of measured, independent and observed [*I* > 2.0s(*I*)] reflections	42213, 1298, 843	42955, 1309, 899	42679, 1483, 1024	42506, 1476, 1018
R_{int}	0.106	0.072	0.072	0.071
q_{max} (°)	20.9	20.9	20.9	20.9
(sin q/l)$_{max}$ (Å^{-1})	0.501	0.501	0.501	0.501
Refinement				
$R[F^2 > 2s(F^2)]$, $wR(F^2)$, S	0.051, 0.137, 0.88	0.042, 0.108, 1.00	0.033, 0.071, 1.00	0.034, 0.099, 1.00
No. of reflections	1229	1219	1427	1424
No. of parameters	87	91	91	92
No. of restraints	135	134	134	144
H-atom treatment	H atoms treated by a mixture of independent and constrained refinement	H atoms treated by a mixture of independent and constrained refinement	H atoms treated by a mixture of independent and constrained refinement	H atoms treated by a mixture of independent and constrained refinement
$D\rho_{max}$, $D\rho_{min}$ (e Å^{-3})	1.78, -0.88	1.46, -0.91	0.66, -0.87	1.27, -0.68

High-pressure data were collected from ambient pressure (on a fiber), then within the DAC at 0.10, 0.23, 0.55, 1.10, 1.40, 1.64 and 2.1 GPa. The coplanar and twisted conformer disorder in the peb^{2-} ligand occurs in such a way, that the C-atoms from each disorder component (labelled C8, C(9), C(10) and C(11) in the structure, Figure S2) sit on one of two mirror planes (Wyckoff position 96g, ..m site). The planar phenyl ring disorder involves C(11) which sits on a mirror, while the twisted phenyl ring

disorder (composed of (C(9) and C(10)) sit on either side of a mirror plane. Both mirror planes bisect each other. On refining the occupancy and thermal parameters for C(9), C(10) and C(11), we determined that the best model was found when these atoms were refined with isotropic thermal parameters, which significantly decreased the number of refined parameters for these partially occupied C-atoms. In addition, libration could clearly be seen in the ambient pressure data set when these were refined with anisotropic displacement parameters, however even here, they are constrained by the bisecting mirror planes to be orthogonal in two directions because of the high site-symmetry (C(9), C(10) and C(11) have only 4-unique displacement parameters). In order to not bias the refinement, and make sure that correlations between the site occupancy of the disorder and thermal parameters did not affect the change in occupancy of the co-planar and twisted conformations observed, we refined the occupancy with isotopic and anistropic displacement parameter models and plotted these against each other (Figure S3). In Figure S3, no statistically significant difference was observed in the crystallographically-determined occupancy. The population of the coplanar (χ_C) and perpendicular (χ_P) is summarised in Table S1. The coplanar structure population as a function of pressure is also depicted in Figure S1 and S2.

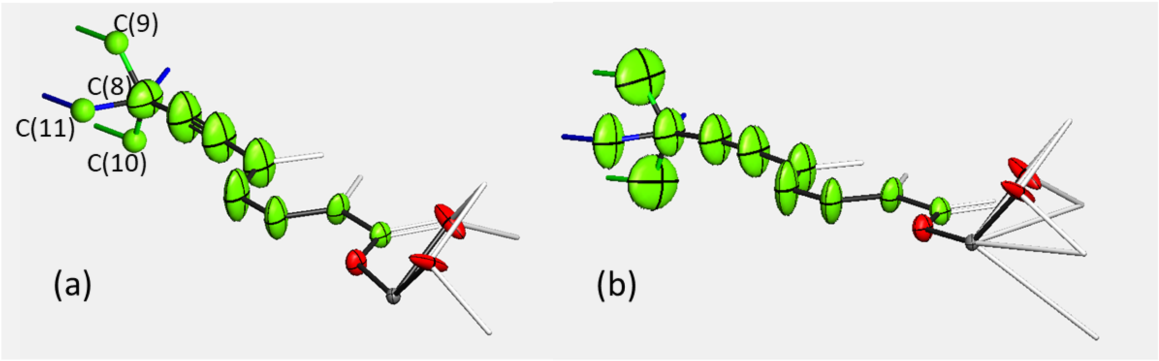

Figure S2. Disordered coplanar and twisted conformers of the peb^{2-} linker as shown in the crystallographic refinement at ambient pressure and temperature, with the disorder modelled with (a) isotropic and (b) anisotropic displacement parameters. Broken bonds indicate bonds formed to symmetry equivalent atoms. Colour scheme C green, O red, Hf grey. H-atoms have been omitted for clarity.

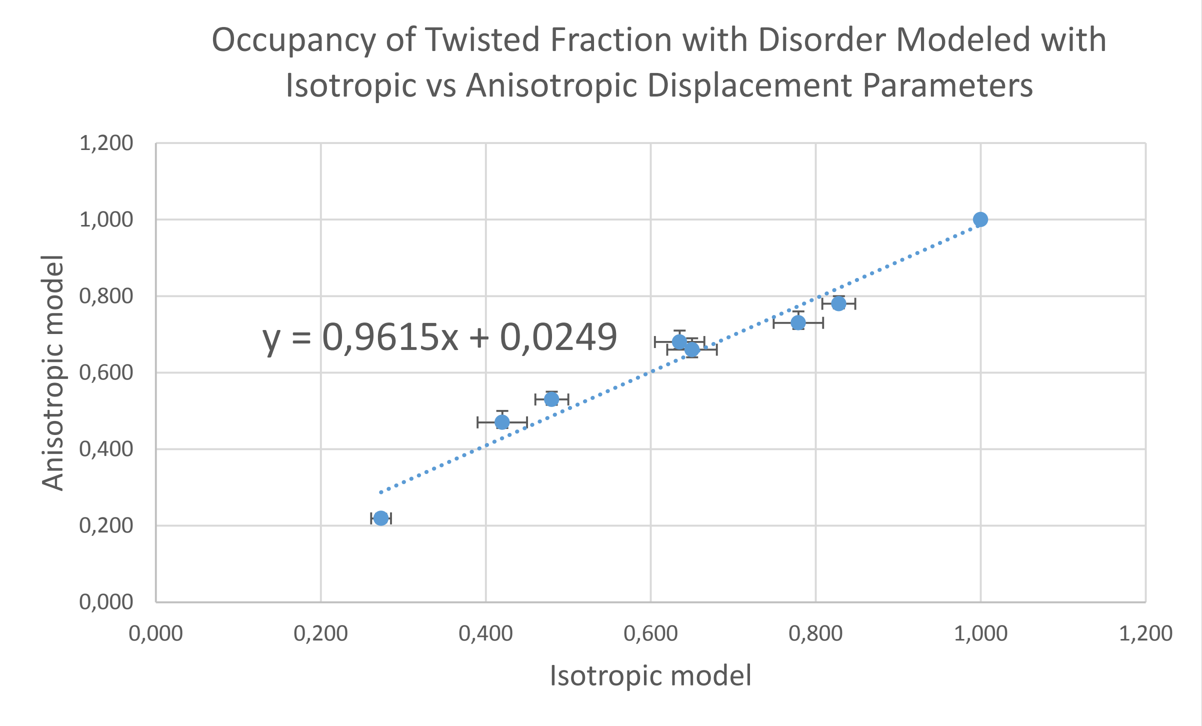

Figure S3. The coplanar and twisted conformers of the peb^{2-} linker occupancies plotted when the C-atoms within the disordered phenyl ring were refined with isotropic and anisotropic displacement parameters.

2. Fluorescence spectroscopy

2.1 Measurement of high-pressure fluorescence emission spectra

Crystals of Hf-peb MOF were placed alongside a ruby chip inside a Merrill-Bassett DAC, with pentane as the hydrostatic medium. A number of crystals were loaded into the DAC to increase the fluorescence intensity. Fluorescence emission spectra were collected at ambient pressure, then at increasing pressures to closely match those used for x-ray diffraction measurements.

Emission spectra were collected using a custom-built spectrometer system, illustrated in Figure S2. A 380-nm LED (Ocean Optics LS-LED) was utilised as the excitation l source. Two plano-convex fused-silica lenses (f 30mm and 10mm) guided the excitation light into a 15X/0.28 infinity-corrected reflective objective lens (ROL) which focused the light into the DAC. The fluorescence from the sample in the DAC was collected using a second ROL and focused, using a second fused silica into a fibre-coupled spectrometer (Ocean Optics USB2000+), controlled by SpectraSuite (Ocean Optics) software. A band-pass filter (Semrock Brightline 370/36) was inserted in the excitation path to narrow the excitation bandwidth. A long-pass filter was inserted in the detection path to prevent the transmitted excitation light from reaching the detector. Fluorescence spectra were corrected for the wavelength-response of the spectrometer.

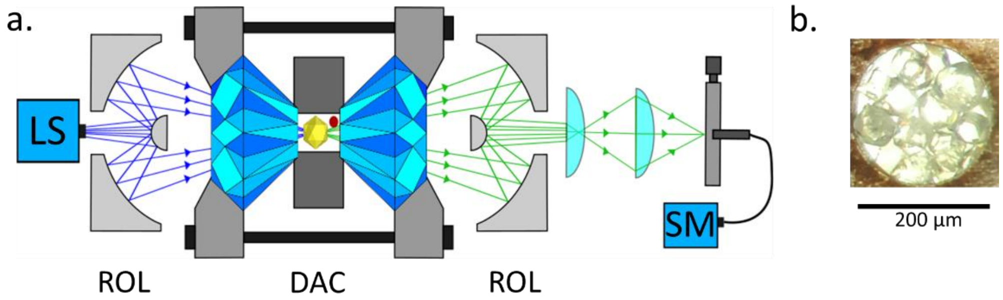

Figure S4 a. Schematic of the spectrometer for high-pressure fluorescence measurements The system consists of a LED light source (LS), reflective objective lenses (ROLs), diamond anvil cell (DAC) and fibre-coupled spectrometer (SM). **b.** photograph of the Hf-peb MOF crystals in the DAC.

The fluorescence spectra are shown in Figure S3 and the wavelengths and wavenumbers of fluorescence maxima are summarised in Table S5.

Table S5. Fluorescence emission maxima as a function of pressure.

Pressure (GPa)	Emission Peak (nm)	Emission Peak (cm^{-1})
**0.00**	428	23360
**0.14**	433	23090
**0.22**	434	23040
**0.38**	438	22830
**0.58**	440	22730
**0.91**	443	22570
**1.29**	443	22570
**1.46**	443	22570
**1.68**	444	22520
**2.12**	450	22220

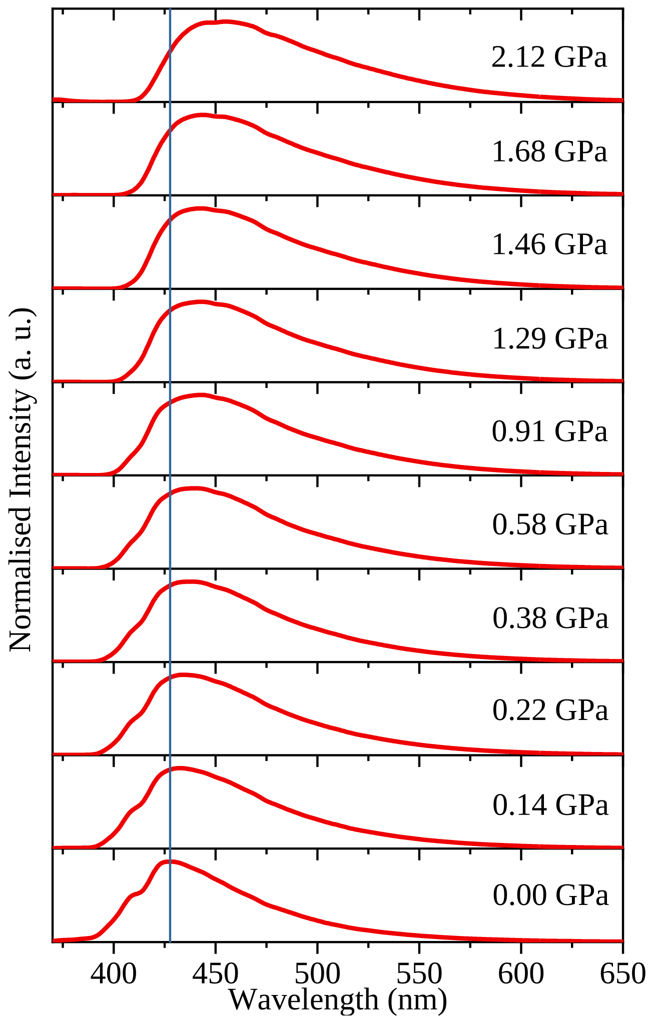

Figure S5. Fluorescence emission spectra of Hf-peb as a function of pressure.

2.2 Fitting of fluorescence spectra

The observed fluorescence spectra were fitted to a linear combination of the 0-GPa (ambient) and 2.1-GPa (twisted conformer) spectra, according to equation (1), using the Solver facility in Excel.

$$S_P \ = \ A_P \, S_0 + B_P S_T \qquad (1)$$

where S_P is the spectrum at pressure P, S_0 is the spectrum at 0 GPa, S_T is the spectrum of the twisted conformer (2.1 GPa), and A_P and B_P are the amplitudes at pressure P.

Given that

$$S_0 = 0.73 S_C + 0.27 S_T \qquad (2)$$

where S_C is the spectrum of the coplanar conformer, the fractional contribution of coplanar and twisted conformers to each spectrum, χ_{PC} and χ_{PT}, respectively, is given by:

$$\chi_{PC} = 0.73 A_P \qquad (3)$$

$$\chi_{PT} = 0.27 A_P + B_P \qquad (4)$$

An example of the spectral fitting is shown in Figure S4.

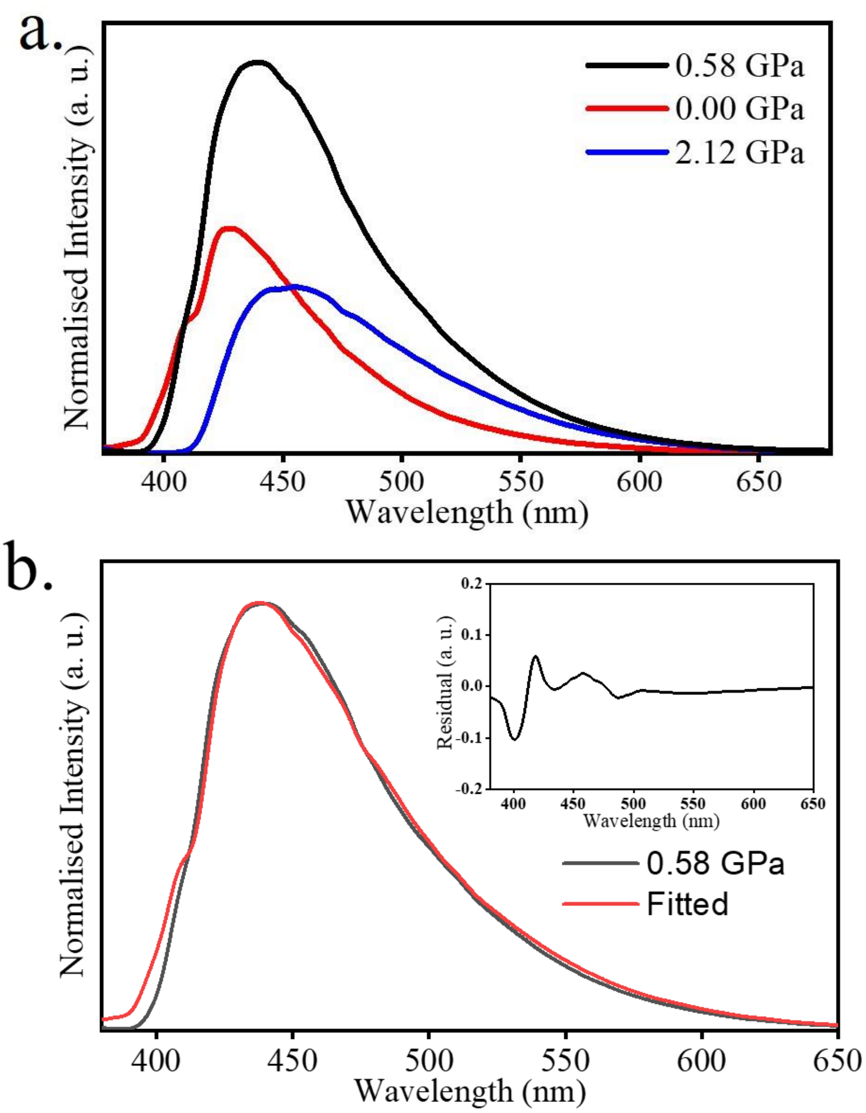

Figure S6 a. The fitted contributions to the 0.58-GPa fluorescence spectrum of the 0-GPa spectral component (red) and the 2.1 GPa spectral component (blue), with fractional amplitudes of 0.61 and 0.39, respectively. **b.** Comparison of the fitted spectrum (red) and measured spectrum (black); the inset shows the residuals.

The fractional contributions of the coplanar and twisted conformations to each spectrum are listed in Table S6.

Table S6. The fractional populations of coplanar and twisted conformers, χ_C and χ_T as a function of pressure, determined from fitting fluorescence spectra.

Pressure (GPa)	χ_C	χ_P
**0**	0.73	0.27
**0.14**	0.57	0.43
**0.22**	0.52	0.48
**0.38**	0.48	0.52
**0.58**	0.44	0.56
**0.91**	0.37	0.63
**1.29**	0.31	0.69
**1.46**	0.25	0.75
**1.68**	0.19	0.81
**2.12**	0.0	1.0

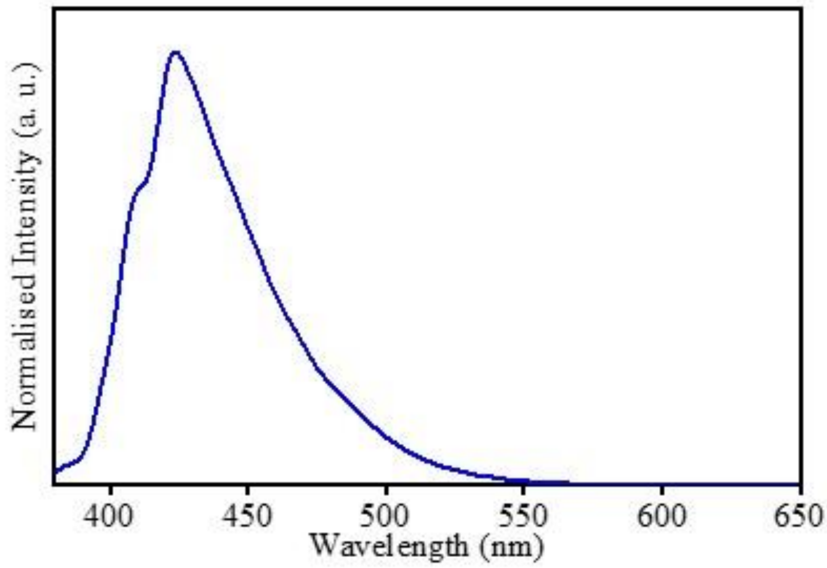

Figure S7. The fluorescence spectrum of the coplanar conformer, extracted from the ambient-pressure spectrum on the basis of the fractional contributions shown in Table S6.

3. Measurement of high-pressure UV-Vis absorption spectra

The experimental system, Figure S6, is similar to that used for the measurement of fluorescence spectra, but uses a broadband deuterium-halogen lamp (Ocean Optics DH-2000-BAL) as the light source and the detection optics are modified to project a magnified image of the sample cell onto the entrance plane of the detection spectrometer. A single crystal of Hf-peb MOF was placed alongside a ruby chip in the DAC, leaving sufficient empty space to measure the transmission of the DAC, in the absence of the sample, as the reference beam. The position of the DAC, and hence the position of the projected image on the detector entrance, was manipulated using a precision 3-axis translation stage

to enable measurement of the reference beam, I_0, and the sample beam, I_S, as illustrated in Figure S5. The transmittance of the sample is then given by $\frac{I_S}{I_0}$.

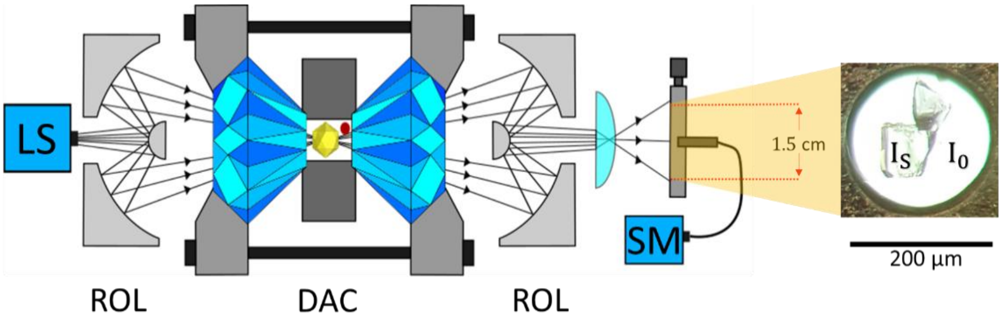

Figure S8. Schematic of the spectrometer for high-pressure UV-Vis absorption measurements. The system consists of a broadband light source (LS), reflective objective lenses (ROLs), diamond anvil cell (DAC) and fibre-coupled spectrometer (SM). A photograph of the Hf-peb MOF crystal in the DAC shows the positions at which reference and sample beams, I_0 and I_S, respectively, are measured.

Absorption spectra were collected at ambient pressure, then at increasing pressures to closely match those used for X-ray diffraction measurements. The wavelength of the absorption edge was determined from the peak of the first derivative of the spectrum.

The absorption spectra are shown in Figure S7 and wavenumbers of the absorption edge are summarised in Table S7.

Table S7. Wavenumber of the UV-vis absorption edge as a function of pressure.

Pressure (GPa)	Absorption Edge (cm^{-1})
**0**	25608
**0.11**	25542
**0.25**	25510
**0.49**	25477
**0.74**	25464
**1.00**	25458
**1.51**	25271
**1.82**	25278
**2.15**	25062

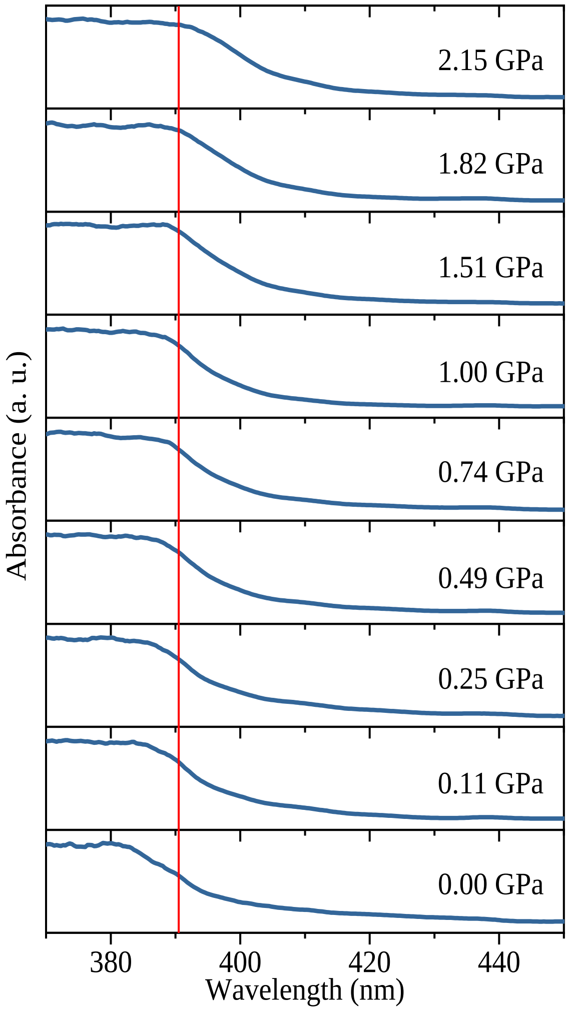

Figure S9. UV-vis absorption spectra of Hf-peb as a function of pressure.

References

[1] S. A. Moggach, D. R. Allan, S. Parsons, J. E. Warren, *J. Appl. Crystallogr.* **2008**, *41*, 249-251.

[2] J. D. Barnett, S. Block, G. J. Piermarini, *Rev. Sci. Instrum.* **1973**, *44*, 1-9.

[3] aB.-A. X. S. Bruker-Nonius, Madison, Wisconsin, USA, **2004**; bS. Parsons, **2004**; cG. M. Sheldrick, **2008**.

[4] P. W. Betteridge, J. R. Carruthers, R. I. Cooper, K. Prout, D. J. Watkin, *J. Appl. Crystallogr.* **2003**, *36*, 1487-1487.
